# Study on acoustic emission characteristics of muddy limestone under brazilian splitting tests at various loading rates

**DOI:** 10.1038/s41598-026-48578-0

**Published:** 2026-04-24

**Authors:** Zhiliang Yang, Bin Liu, Donghui Yang

**Affiliations:** 1 School of Coal Engineering, Shanxi Datong University, Datong, 037003 Shanxi China; 2 The Cultivation Base of Shanxi Key Laboratory of Coal Mine Water Jet Technology and Equipment, Shanxi Datong University, Datong, 037003 Shanxi China; 3 State Key Laboratory of Digital Intelligent Technology for Unmanned Coal Mining, Anhui University of Science and Technology, Huainan, 232001 Anhui China

**Keywords:** Muddy limestone, Loading rate, Tensile strength, Acoustic emission characteristics, Fractrue, Engineering, Materials science, Solid Earth sciences

## Abstract

To explore the correlation between Acoustic Emission (AE) and fracture response characteristics and loading rates in the failure and instability of muddy limestone, the borehole near the Baode Coal Mine Power is taken as the research object, Brazilian splitting tests combined with acoustic emission measurements were conducted on muddy limestone specimens at various loading rates, mechanical parameters, AE phenomena, and fracture response features of muddy limestone were analyzed. The results demonstrate the average tensile strength, elastic modulus, peak strain, and residual strength of the rock specimen exhibit stage-dependent loading effects. At low loading rates, ample deformation and damage occur within the rock, resulting in more severe fragmentation, with increasing loading rate, rock specimens gradually evolve from shear-tensile failure dominated by shear-tensile mechanisms to tensile failure.As the loading rate increases, distinct AE signals occur earlier, both cumulative ring count and cumulative energy first increase and then decrease, and the AE energy changes from isolated event type to cluster event type.The AE b-value shows a gradual decreasing trend.

## Introduction

The tensile strength of rock is significantly lower than its compressive strength, and tensile failure is common in rock mechanics^1^. The tensile strength of rock is essential for the structural design and stability assessment of deep underground engineering. Therefore, testing for tensile strength is an active area of study^2^. The tensile strength of rock is influenced by inherent factors, including rock type^3^, mineral composition^4^, sedimentary age^5^, burial depth^6^, cementation type^7^, and bedding structure^8^, as well as loading rate. The speed of excavation, the method used, and the spatial dimensions of engineering projects all influence the loading rate on the surrounding rock. This, in turn, affects the rock’s splitting characteristics due to loading rate effects, which impacts the stability of rock mass engineering^9^.Thus, investigating various loading rates offers significant theoretical and engineering value.

Numerous scholars have conducted extensive research on rock splitting tests under various loading rates, yielding substantial findings. Chen Ke et al.^10^ investigated the AE characteristics of sandstone under various loading rates during Brazilian splitting tests, revealing that both AE ringing and energy levels increase with the loading rate. Ren Song et al.^11^ conducted Brazilian splitting tests on coal at different loading rates, showing that the probability density distribution of acoustic emission energy follows a probabilistic distribution. Wang Cheng et al.^12^ concluded, based on Brazilian splitting test results of limestone at varying loading rates, that splitting strength and splitting energy rate have a logarithmic relationship with loading rate. Li Xiaolong et al.^13^ investigated the impact of loading rate on AE during Brazilian splitting of weakly cemented sandstone.They found that the surge point of AE counts corresponds with the peak stress point, indicating that a significant number of AE events occur at the moment of rock failure. Wei Sijiang et al.^14^ conducted Brazilian splitting tests on both anchored and unanchored coal samples under static loading conditions, with varying rates of loading. Their findings revealed that the effect of loading rate on the tensile strength and final splitting energy of coal samples is generally consistent across both types. Notably, the anchored samples consistently exhibited higher tensile strength and final splitting energy compared to the unanchored samples. Chen Yan et al.^15^ conducted research on Brazilian splitting tests involving coal-rock composites at various loading rates. Their findings reveal a strong positive correlation between tensile strength and loading rate. Specifically, at lower loading rates, there is a significant variation in the tensile strength of the specimens. However, this increase in strength diminishes as the loading rate increases. This insight is crucial for understanding how loading conditions affect the performance of coal-rock composites, highlighting the importance of considering loading rates in material testing and application. Peng et al.^16,17^ conducted true triaxial acoustic emission tests on red sandstone specimens containing an arched-hole, revealing that both the cumulative acoustic emission counts and the absolute energy release rise as the minimum principal stress increases.

Several studies have analyzed how loading rates affect the strength of various types of rocks, including sandstones, limestones, coal, and other minerals. However, research specifically focusing on the strength of Carboniferous muddy limestone is scarce. This gap in the literature presents an opportunity for further investigation that could yield valuable insights into the behavior of this unique rock formation. Due to the limited research on the effect of loading rate on Carboniferous muddy limestone, Brazilian splitting-acoustic emission tests were conducted at various loading rates. In this study, the mechanical parameter, the AE characteristics, and the failure modes were analyzed in relation to loading rate. The results, particularly the evolution of AE parameters such as ring count, energy, and b-value, offer valuable insights for early warning of rock instability. Additionally, these findings can help optimize coal mining extraction rates, ensuring both safety and efficiency in the mining process.

## Specimens and experimental methods

### Specimen preparation

**Fig. 1:**
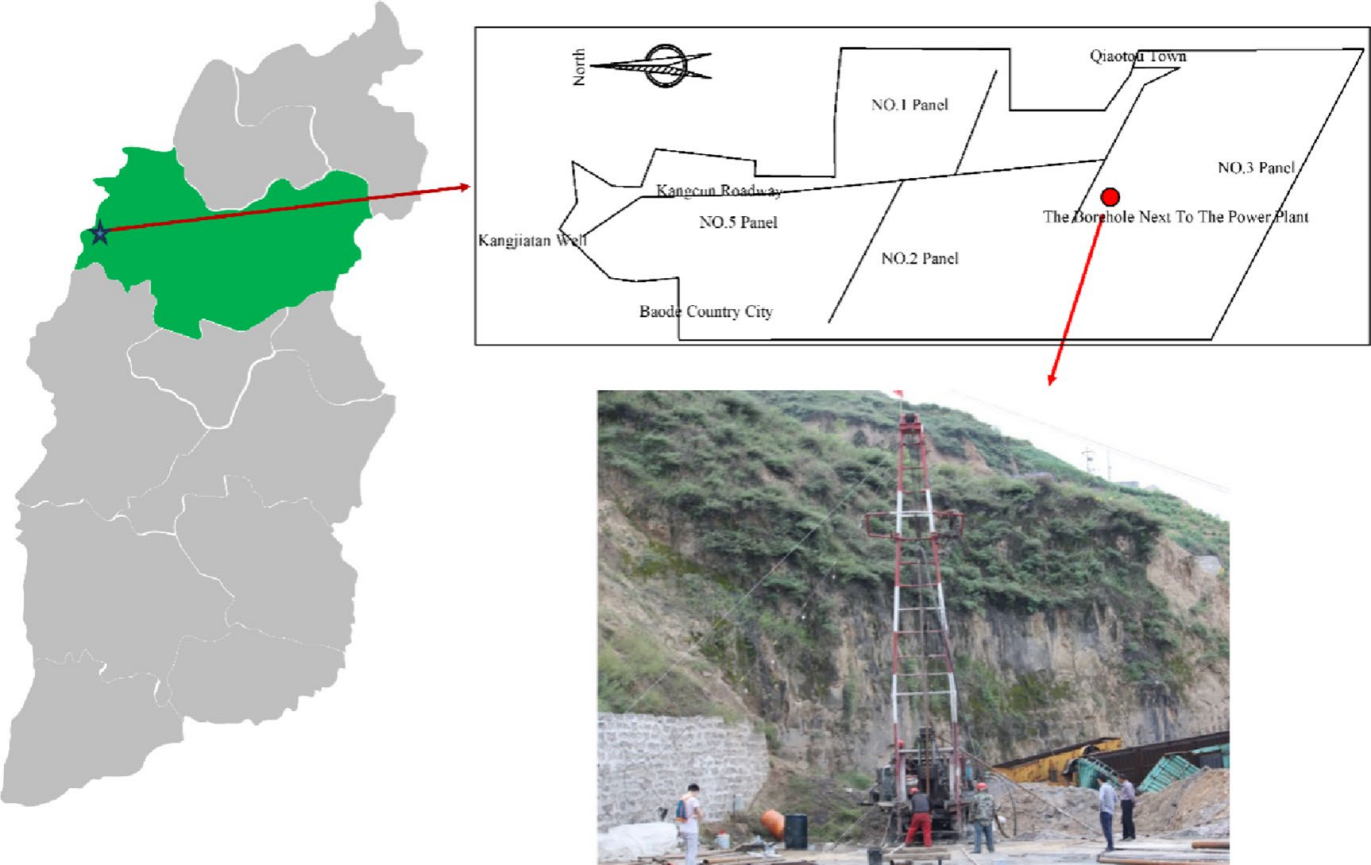
The location of the drilling nearby Baode Mine power plant.

**Fig. 2:**
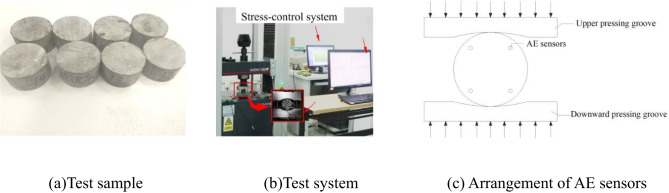
Test samples, test system and arrangement of AE sensors. (**a**) Test sample, (**b**) Test system, (**c**) Arrangement of AE sensors.

The experimental rock cores were collected from a geological borehole near the Baode Coal Mine Power Plant, Shan Xi Province, China. This borehole, which reaches a depth of 416.35 m, has been instrumental in investigating the geological and engineering characteristics of the surrounding strata. The location of the drilling hole is shown in Fig. 1. Permission was obtained for collection of rock samples from mines, and the muddy limestone cores were collected from a depth of 416 m. Based on geological data, this muddy limestone is characterized by a dark black color, particle sizes of less than 0.01 mm, shell-like fracture surfaces, a semi-hard consistency, and well-developed horizontal bedding. To prevent weathering, the extracted rock cores were wrapped in plastic and stored in core boxes. In accordance with the International Society for Rock Mechanics (ISRM)^18^ standards, the cores were processed by drilling, cutting, and grinding into discs with a diameter of 50 mm and a height of 25 mm. Four groups were selected based on different loading rates: 0.05, 0.20, 0.50, and 1.00 mm/min. Each group consisted of several samples, and the processing precision met the experimental requirements, as shown in Fig. 2(a). The corresponding physical and mechanical parameters of the specimens at various loading rates are detailed in Table 1.

**Table 1:** Physical and mechanical parameters.

Loading Rates (mm.min^− 1^)	Specimens	Diameter (mm)	Height (mm)	Tensile Strength(MPa)	Elastic Modulus (GPa)	Residual Strength (MPa)	Peak Strain (10^− 3^)
0.05	A1	49.04	25.32	16.27	1.79	1.51	11.64
	A2	48.72	25.56	19.40	1.64	1.18	14.68
	A3	49.24	25.16	18.24	1.78	0.59	14.26
0.20	B1	49.04	25.40	12.53	1.75	2.60	11.20
	B2	48.52	26.87	22.29	1.67	1.63	17.87
	B3	49.26	25.88	21.56	1.96	0.73	13.83
0.50	C1	48.95	25.18	12.37	1.81	2.82	11.06
	C2	49.01	25.26	18.57	2.00	1.28	13.56
	C3	49.00	25.53	18.00	2.04	1.32	12.43
1.00	D1	48.66	26.30	14.37	1.38	4.00	12.27
	D2	48.40	25.39	12.42	1.56	5.61	10.38

### Experimental equipment

The electronic universal testing machine (Model: WDW-100E) and AE monitoring system (Model: PCI-2) were employed to conducted Brazilian splitting tests on specimens, as shown in Fig. 2(b). The test system is capable of recording load-displacement data in real time, with a maximum load capacity of 100 kN and a load rate range of 0.005 to 500 mm/min. Additionally, the AE signals generated during the damage and failure processes are simultaneously captured using the PCI-2 device from Physical Acoustic Corporation, operating in waveform streaming mode. The AE testing system comprises four Micro Nano30 sensors and a six-channel preamplifier. It offers a frequency bandwidth ranging from 1 kHz to 3 MHz, with a maximum signal amplitude of 100 dB and a dynamic range exceeding 85 dB. This advanced setup ensures precise and reliable monitoring for testing needs. Additionally, arc-shaped fixtures were precisely machined for splitting tests. Ensuring no personnel movement during testing to eliminate interference with the AE system.

### Test scheme

The test utilized arc-shaped fixtures for the splitting tests. As shown in Fig. 2 (c), four sensors are evenly pasted on the specimen’s surface using vaseline. The central frequency of the Nano30 sensor is 140 kHz, and the frequency range from 125 to 750 kHz. After repeated measurements of environmental noise, the threshold and the acquisition frequency were set to 45 dB and 1 MHz, respectively. To analyze the impact of loading rate on mechanical properties, a displacement-controlled loading approach was used, and four loading rates of 0.05, 0.20, 0.50, and 1.00 mm/min were conducted. During the test, both the AE monitoring system and test system were activated simultaneously, with all parameters recorded. The indirect tensile strength of the specimen can be calculated using the following Eq.^19^.

$$\:{R}_{t}=\frac{2P}{\pi\:Dh}$$

Where *R*_t_ is the tensile strength of the specimen(MPa), *P* represents the failure load(kN), while D and h denote the diameter and height of the specimen(mm), respectively.

## Results analysis

### Stress-strain curve

**Fig. 3:**
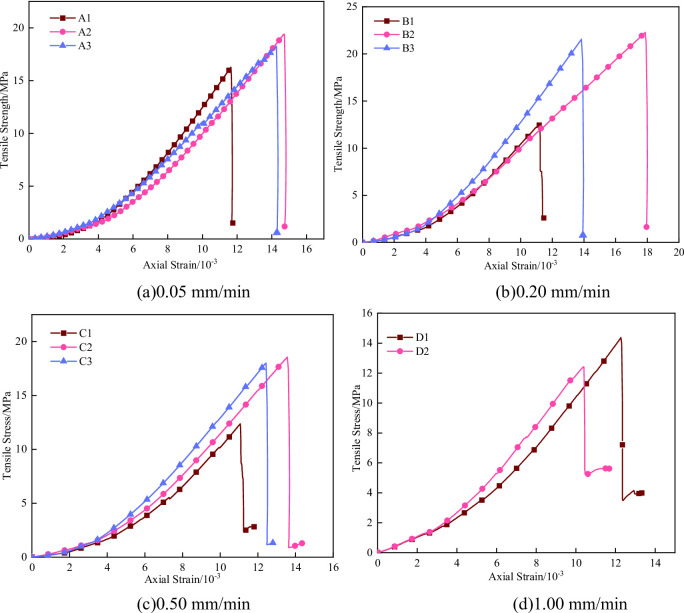
The curves of tensile stress-strain for various loading rates. (**a**) 0.05 mm/min, (**b**) 0.20 mm/min (**c**) 0.50 mm/min, (**d**) 1.00 mm/min.

The stress-strain curves for various loading rates are shown in Fig. 3. It can be observed that the stress-strain curve can be divided into four distinct stages: compaction, elastic, plastic, and failure. Muddy limestone shows clear brittle behavior at different loading rates, marked by a swift decline after peak stress. The bearing capacity significantly decreases at low loading rates (0.05 and 0.20 mm/min), while a rebound effect is observed at higher loading rates (0.50 and 1.00 mm/min). As the loading rates increase, the specimen does not have enough time to deform, resulting in enhanced stiffness and, consequently, an increased bearing capacity. In general, the total average strain at low loading rates is greater than at high loading rates. Specifically, the strain measures 13.60 × 10^− 3^ for 0.05 mm/min and 14.45 × 10^− 3^ for 0.20 mm/min, compared to 12.97 × 10^− 3^ for 0.50 mm/min and 12.51 × 10^− 3^ for 1.00 mm/min. This difference occurs because, at low loading rates, the rock has sufficient time to deform, allowing cracks to fully develop. In contrast, at high loading rates, failure happens before the rock can completely fracture.

### Mechanical parameter characteristics

**Fig. 4:**
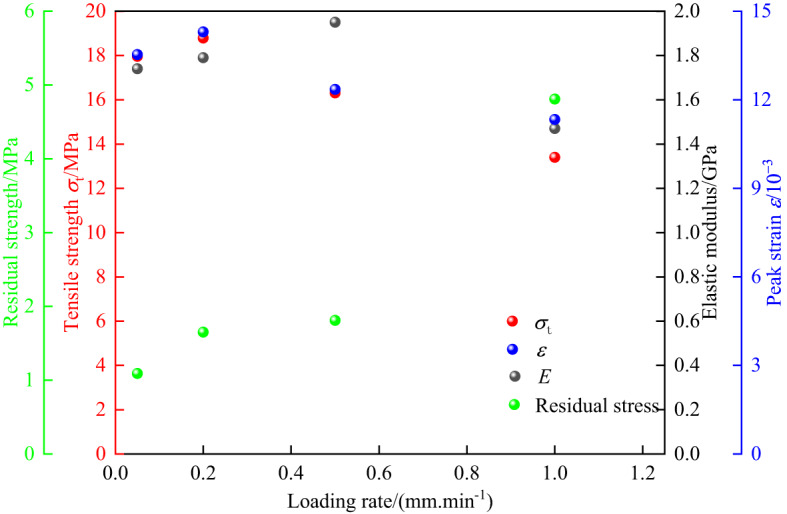
Relationship between the tensile strength, modulus, residual strength, peak strain of the specimen and various loading rates.

The relationships among average tensile strength, residual strength, elastic modulus, and peak strain with loading rate are illustrated in Fig. 4. It is apparent that the average tensile strength of A, B,C, and D groups are 17.86, 19.24, 16.20 and 13.40 M, respectively, and the coefficient of dispersion are 8.94%, 31.29%, 21.40%, and 13.18%, respectively .The reason for the discreteness of each set of data may be that uniform and identical specimens were not screened out through wave speed before the test. The figure indicates that the average tensile strength and peak strain follow a consistent trend, both initially increasing and subsequently decreasing with respected to increasing loading rates, increasing by 4.62% and 5.69%, respectively, with increasing loading rates from 0.05 mm/min to 0.20 mm/min, decreasing by 14.16% and13.63% respectively, with increasing loading rates from 0.20 mm/min to 0.50 mm/min, and further decreasing by 17.84% and 8.26% respectively with increasing loading rates from 0.50 mm/min to 1.00 mm/min.

The mean elastic modulus displayed a clear trend of initially increasing and then decreasing as loading rates increased. Specifically, it rose by 2.87% when the loading rate was increased from 0.05 mm/min to 0.20 mm/min. It further increased by 8.94% when the loading rate was elevated from 0.20 mm/min to 0.50 mm/min. However, this was followed by a significant decrease of 24.61% when the loading rate was adjusted from 0.50 mm/min to 1.00 mm/min.

It can be observed that the average tensile strength, peak strain, and elastic modulus demonstrate loading rate effects. When the loading rate surpasses a specific critical value, the average tensile strength and elastic modulus tend to stabilize or even decrease.This observation aligns with conclusions reported in existing literature^20^. Notably, the critical loading rate for average tensile strength is lower than that for the elastic modulus.

As illustrated in Fig. 4, the residual strength shows a marked increase with higher loading rates. Specifically, it rises significantly by 51.38% when the loading rate increases from 0.05 mm/min to 0.20 mm/min. Following this initial surge, the increase becomes more gradual, with only a slight rise noted as loading rates progress from 0.20 mm/min to 0.50 mm/min. Importantly, the growth pattern appears to be approximately exponential, leading to a remarkable increase of 165.74% when the loading rates are elevated from 0.20 mm/min to 0.50 mm/min. Marlstone has a closely arranged microstructure and is relatively compact overall. It consists of many irregularly shaped particles that are rough, with a particle size of less than 0.01 mm. The primary component is calcite, along with some viscous minerals. Under low loading rates, cracks become fully developed, the edges of the particles are worn down, and the spaces between the particles increase. Additionally, the expansion of clay minerals raises the volume, which exacerbates the damage to the marl. In contrast, under high loading rates, the cracks do not fully develop, the wear on the particles occurs over a shorter time, and the internal density of the marl remains better. As a result, the degree of damage is smaller in this case.

## AE characteristics for various loading rates of specimen

### AE ringing count characteristics

**Fig. 5:**
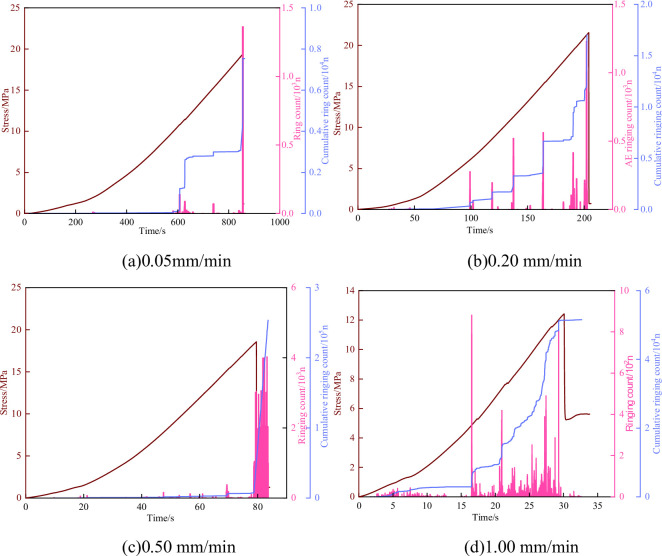
The stress-AE ring count-time distribution characteristics for various loading rates. (**a**) 0.05 mm/min, (**b**) 0.20 mm/min, (**c**) 0.50 mm/min, (**d**)1.00 mm/min.

Figure 5 illustrates the distribution characteristics of stress-AE ringing count-time. In the Fig. 5(a), in the case of lower loading rate of 0.05 mm/min, less cumulative AE are detected, and a longer time needed for the entire failure of the specimen. It can be observed that AE is weak during the initial loading phase, the cumulative ring count exhibits negligible growth, and the steady growth stage continues for approximately 590 s. As axial stress loading increases, the extent of internal local fracture propagation become more pronounced, leading to heightened AE activity. The cumulative ring count curve indicates an initial increase in activity, although the rise is not substantial, remaining below 0.12 × 10^4^ times. During the plastic deformation phase, crack propagation accelerates, leading to an increase in AE activity. The cumulative ring count rises steadily but shows a significant spike at 855 s. At this point, the rock specimen is approaching fracture, characterized by rapid crack propagation and heightened AE activity. Ultimately, the final cumulative ring count reaches 0.75 × 10 ^4^ times.

As illustrated in Fig. 5(b), at higher loading rates, the cumulative ring counts demonstrate no significant increase during the initial loading phase. However, a slight stepwise rise occurs at 69.9 s, reaching a ring count of 0.63 × 10^4^ counts. This is followed by a period of steady growth. As the specimen approaches failure at 78.8 s, the AE enters a burst growth stage, and the cumulative ring counts escalate dramatically to reaching 25.35 × 10^4^ times.

As the loading rate increases to 1.00 mm/min, as shown in Fig. 5(d), the growth rate of the cumulative ringing counts accelerated significantly.This increase is accompanied by a decrease in the number of steps and an increase in the step amplitude. Notably, the ring count occurs a sudden spike occurs at 17 s, and the cumulative ring counts reach 5.15 × 10^4^ times.This phenomenon is attributed to the decrease in tensile strength and crack initiation resistance under high loading rates, leading to the earlier occurrence of more pronounced AE signals.

### AE energy characteristics

**Fig. 6:**
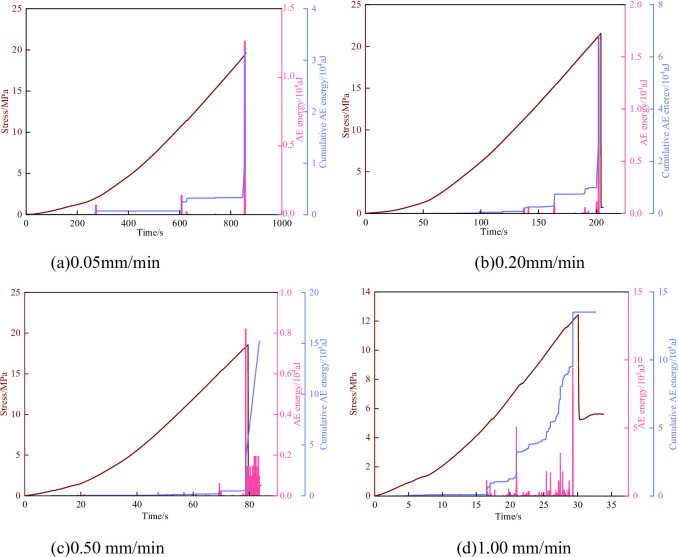
The stress-AE-time distribution characteristics for various loading rates. (**a**) 0.05 mm/min, (**b**) 0.20 mm/min, (**c**) 0.50 mm/min, (**d**) 1.00 mm/min.

Figure 6 illustrates the stress-AE energy-time distribution characteristics, it can be seen that the variation trends of AE energy and AE ring count are similar, indicating a strong correlation among AE characteristic parameters. Based on seismic wave energy analysis, the AE energy release characteristics include mainshock type, cluster type, and isolated event type^21^.

As shown in Fig. 6(a), at relatively low loading rate (0.05 mm/min), the fracture propagation within the rock specimen is gradual, the AE activity is weak, and the overall AE energy reflects isolated event type. During the compaction and elastic stages, the cumulative energy progresses in a stepped manner, with energy fluctuations primarily occurring before and after instability failure. This culminates in a peak energy level, with a final cumulative energy of 3.14 × 10 ^4^ aJ.

As shown in Fig. 6(b), at moderate loading rate(0.20 mm/min), the crack propagation rate accelerates, leading to intensified activity. Notably, the amplitude density of AE capability significantly increases prior to failure. Moreover, the cumulative energy reaches 15.21 × 10^4^ aJ, indicating a striking increase of 384.39% compared to lower loading rates.

As illustrated in Fig. 6(d), at higher loading rates (1.00 mm/min), the AE energy and density significantly increase, demonstrating a clustered distribution of event. The trend of cumulative energy growth closely resembles that of cumulative ringing evolution, with AE cumulative energy of 13.51 × 10 ^4^ aJ, showing no significant difference from the condition at 0.50 mm/min.This analysis indicates that a larger portion of the rock’s energy is utilized in fracturing, resulting in relatively less energy being converted into AE energy.

### Analysis of fracture characteristics

**Fig. 7:**
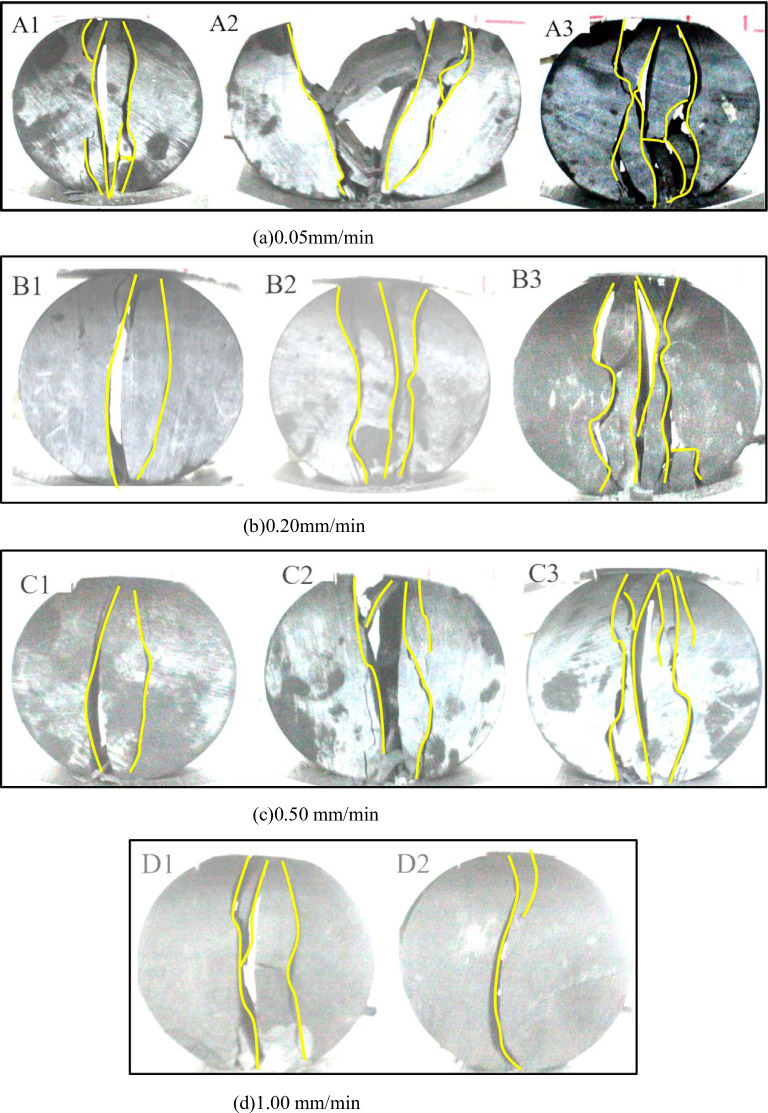
Failure morphology of the specimen for various loading rates. (**a**) 0.05 mm/min, (**b**) 0.20 mm/min, (**c**) 0.50 mm/min, (**d**) 1.00 mm/min.

The failure modes of the specimens under various loading rates are illustrated in the Fig. 7. Under various loading rates, the specimens exhibited one or more primary cracks. In the Fig. 7(a), at a loading rate of 0.05 mm/min, cracks originated from the disk center, leading to the formation of multiple primary crack. This created tears along the direction of these cracks, resulting in significant block detachment and severe failures of the specimen. In Fig. 7(b), at a loading rate of 0.10 mm/min, cracks initiated from both the upper and lower loading jaws. This resulted in the formation of 2 to 3 arc-shaped primary cracks, accompanied by small blocks and powder detachment at the ends. These observations indicate a significant failure of the specimen. In the Fig. 7(c), at a loading rate of 0.50 mm/min, the primary initiates at the loading point and follows an arc-shaped path. This crack deviates from the center of the disc and penetrates deeper. Furthermore, noticeable secondary cracks appear on both sides. Small block detachments at these points indicate significant damage at both ends. In Fig. 7(d), at a loading rate of 1.00 mm/min, the primary crack initiates at the loading point, resulting in a single, relatively deep arc-shaped main crack. While there are no significant secondary cracks at the ends, minor block detachments do occur. Overall, the specimen remains relatively intact.

With increasing loading rate, specimen failure occurs more rapidly, and the extent of internal damage decreases. At low loading rates, internal cracks have sufficient time to propagate and penetrate, resulting in more complete rock failure. The greater the damage to the test specimen, the higher the proportion of shear failure, which consequently results in a lower residual strength. It can be concluded that with increasing loading rate, the failure mode of the specimen transitions from shear-tensile failure, primarily influenced by tensile stresses at low loading rates, to predominantly tensile failure.

### AE b value characteristics

The b^22,23^ value of AE is a crucial parameter that characterizes fractures based on the magnitude-frequency relationship. An increase in the b-value suggests a higher proportion of small events, which are predominantly caused by small-scale microfractures. Conversely, a constant b-value indicates that the distribution of small and large acoustic emissions remains unchanged, reflecting a relatively stable distribution of microfracture scale. A decrease in the b-value, on the other hand, signifies a greater proportion of larger events and the presence of more extensive, large-scale microfractures. Understanding these variations is essential for interpreting the behavior of fractures and the underlying mechanisms at play.

1 $${\text{lg N }} = {\text{ a}} - {\mathrm{bM}}$$

Where N represents the number of AE exceeding the magnitude M, magnitude M = A/20^24^, A is the AE amplitude, a and b are constants.

AE b value based on maximum likelihood estimation^25^

2 $${b_i}=\frac{{\log \left( e \right)}}{{\overline {M} - {M_{\hbox{min} }}}}$$

Where $\:\stackrel{-}{M}\:$is the mean magnitude, *M*_min_ is the minimum magnitude.

**Fig. 8:**
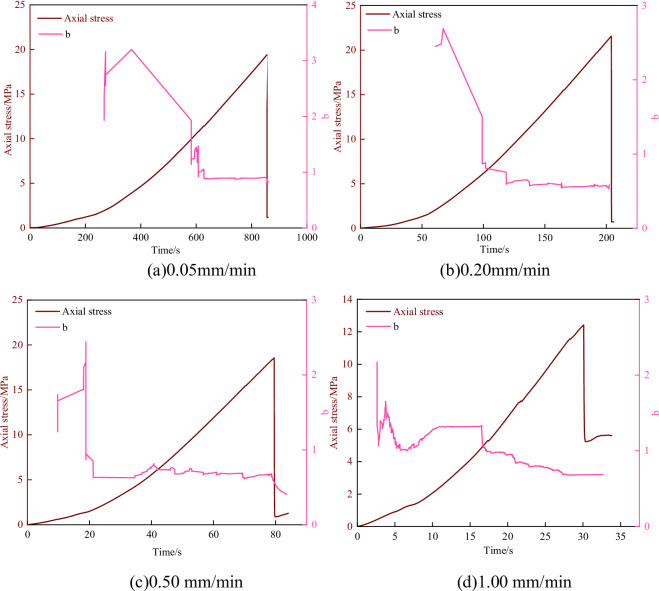
The stress-b value-time distribution characteristics for various loading rates. (**a**) 0.05 mm/min, (**b**) 0.20 mm/min, (**c**) 0.50 mm/min, (**d**) 1.00 mm/min.

Figure 8 illustrates the distribution characteristics of stress-b value-time. It is evident that the b value shows a decreasing trend. At low loading rates of 0.05, 0.20, and 0.50 mm/min, the b value initially increases, then fluctuates, and finally declines. In contrast, at a high loading rate of 1 mm/min, the b value experiences a sharply declines followed by a downward fluctuation, resembling a ‘fish-tail’ pattern. With increasing of loading rates, the ascending phase of the b value during the compaction phase gradually shortens, and the initial b value decreases, indicating that the early compression stage is dominated by the initiation of small-scale fractures, while higher stress induces the expansion of small-scale fractures into large-scale fractures. During the elastic deformation phase, the b value exhibits pronounced fluctuations and begins to decrease, reflecting frequent propagation and coalescence of internal fractures at various scales. During the plastic deformation phase, as the applied stress increases, numerous microcracks aggregate and coalesce to form large-scale fracture surfaces, releasing substantial strain energy. Consequently, the b value enters a declining phase. The maximum value of the b parameter demonstrates a significant negative correlation with the loading rate, declining from 3.2 to 2.90, then to 2.44, and finally to 1.73. This trend clearly indicates an increased intensity of crack propagation within the rock specimen.

### Both RA and AF of AE characteristics

The parameters derived from acoustic emission (AE) metrics, specifically the RA (rise time/amplitude) ans the AF(AE ring count/duration time), can be effectively utilized to distinguish between tensile and shear failure. Generally, AE signals exhibiting a high AF value and a low RA value are indicative of tensile failure. Conversely, a low AF value combined with a high RA value suggests shear failure. Additionally, signals with both low RA and low AF values can be interpreted as indicative of mixed tensile-shear failure^26–31^. Understanding these parameters is crucial for accurate failure analysis and can greatly enhance our ability to predict material behavior under stress.

**Fig. 9:**
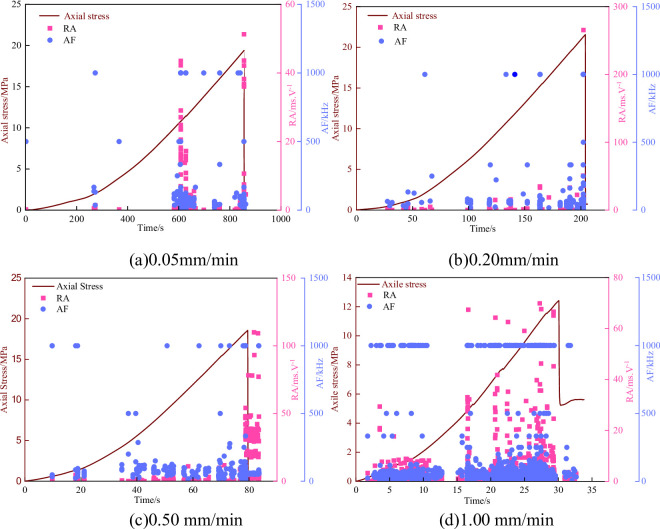
The stress-RA-AF-time distribution characteristics for various loading rates. (**a**) 0.05 mm/min, (**b**) 0.20 mm/min, (**c**) 0.50 mm/min, (**d**) 1.00 mm/min.

Figure 9 presents the rock specimen stress–RA–AF–time distribution characteristics. It can be seen that the variation pattern of the RA-AF values is consistent with the macroscopic fracture.

During the compaction phase, the AF values exhibit a positive correlation with the loading rate, and fracture propagation is primarily influenced by tensile action. In the initial stage of elastic deformation, the RA values exhibit a negative correlation with the loading rate, while the AF values show a positive correlation, indicating a transition of the rock specimen from shear fracture to tensile fracturing. During the elastic deformation development stage, both the AF and the RA values increase, microcracks rapidly propagate, the macroscopic main fracture gradually penetrates, and the transitions from a single fracture to a composite mode of tensile-shear fracturing. During the plastic deformation-failure stage, as the loading rate increases, the RA values relatively decrease, AF values relatively increase, and the final fracture mode transitions from shear to tensile. From the analysis above, it can be concluded that as the loading rate increases, the fracture mode gradually shifts from shear-tensile failure dominated by tensile stress to tensile failure.

## Conclusions

(1) The average strength, elastic modulus, peak strain, and residual strength of the rock specimen exhibit stage-dependent loading effects. The average strength and elastic modulus initially increase and then decrease with increasing loading rate, whereas the residual strength increases as the loading rate increases.

(2) The evolution patterns of AE parameters at different loading rates are consistent, exhibiting phased growth characteristics. As the loading rate increases, distinct AE signals occur earlier, cumulative ring count and cumulative energy first increase and then decrease, with the energy release characteristics transitioning from isolated event type to cluster event type.

(3) Loading rate has a significant influence on the fracture mode and fragmentation morphology of muddy limestone. With increasing loading rate, rock specimens gradually transition from shear-tensile failure dominated by shear-tensile mechanisms to tensile failure. At low loading rates, ample deformation and damage occur within the rock, resulting in more severe fragmentation. The b value exhibits a decreasing trend, under the loading rates of 0.05, 0.20, and 0.50 mm/min, the b value first increases, then fluctuates, and finally decreases, while under the high loading rate of 1 mm/min, the b value sharply declines and subsequently fluctuates downward in a ‘fish-tail’ pattern.

## Data Availability

The mechanical parameters and acoustic emission data of the specimens in the manuscript were obtained in the Mechanics Laboratory of Beijing Computing Center and are available. If the readers want to request the experimental data of this study, they can contact the author Zhiliang Yang, whose email is 1593791548@163.com.
